# Correspondence

**Published:** 1855-01

**Authors:** 


### Correspondence.

CAMBRIDGE, Mass., November 10th, 1854.

JAMES TAYLOR, M. D.

DEAR SIR:—In the October number of the Register, mention is made of "non-contractability of material," in reference to my style of Mineral Plate Teeth. I would correct the supposition that I use a base, or tooth body peculiar for its non-contractability.

I have used porcelain material compounded according to various recipes, and I am persuaded that any porcelain material which will make good block work, will also make this style of work. I have not tried to get a material that will not shrink, but I have tried to obviate the difficulty arising from shrinkage, *and I have succeeded.*

When I have a plaster cast which is a perfect representation of that part of the mouth to be filled, it is cut apart and the pieces separated such distance as corresponds to the amount of shrinkage in the porcelain caused by fusion.

Different compositions will of course differ in the amount of shrinkage, but this, if not known, may be ascertained by an experiment.

This way of enlarging the cast to meet the contraction, answers perfectly every practical requirement; and thus we are enabled to make half or whole sets of teeth entirely of mineral. And on account of the superiority of such sets,—their simplicity and purity, the time and labor saved and the consequent reduction in the manufacture, there is a physical certainty that this method will be adopted ; for it is evidently preferable to wear, being so absolutely clear and free from taste; and, in fact, I know of no relation wherein this is not superior.

To a person who has had no experience in making mineral teeth, it may at first seem difficult to make a set of these, but when the skill has been acquired to do it, it is easier to make a set in this, than in any other way ; and to a person wholly unskilled in making sets of teeth, it is easiest to acquire the art of making them. MAHLON LOOMIS.

---

Dr. TAYLOR.

Please insert the following notice in the January number of the Register.

IMPROVEMENTS.

To the members of the Dental Profession and those engaged in the fabrication of articles used in the practice of dentistry.

The undersigned, chairman of a committee appointed by the Mississippi Valley Association to note and report improvements made during the current year appertaining to the practice of dentistry so far as the same may be made known, together with the names of inventors or discoverers, is desirous of corresponding with those gentlemen who may have made any improvements they consider worthy of note, or discovered principles or things worth the attention of the association.

If the improvement is instrumental or the discovery or principle is of such a nature as to be illustrated by specimens or drawings these should accompany the description.

Specimens and illustrations will be preserved in the society's cabinet or returned if preferred.

The period for making the report is the 20th of February 1855, communications should reach here before that time.

Address A. M. Leslie, Cincinnati, O.

---

Logan, Hocking Co., O. July 7th, 1854.

Mr. Editor:

I wish to call your attention to a preparation I use for soldering—which so far as my knowledge extends is not used by the Profession any where, and which I have found in my own practice of immediate value. Should you deem it worthy of consideration, please give it a place in the Register and thereby perhaps add something to the mighty structure you are laboring so zealously to rear.

It is composed of equal parts of Borax, Saleratus and common salt, well pulverized and mixed.

Manner of Using.—I do not dispense with the old mode of grinding the Borax and applying as usual. After applying the ground *Borax* and *solder*, sprinkle on an indefinite quantity of the above compound. This keeps the plate perfectly clean and affords a very high heat, and also causes the solder to flow with greater ease to any desired point. Any one giving this a fair trial will not in my humble opinion regret the experiment.* Very Respectfully Yours,

B. A. Rose.

*The above was received in July, but too late for insertion in that number, and it escaped our recollection for the October number, and we only laid our hands on it in time for the present number.—[Ed.

